# Marriage intention and influencing factors of ethnic minority residents in Zang-Qiang-Yi Corridor: a perspective based on the Andersen’s behavioral model

**DOI:** 10.3389/fpsyg.2025.1588247

**Published:** 2025-11-07

**Authors:** Lin Cai, Denghui Lu, Juan Ning

**Affiliations:** 1 Institute of Chinese National Community, Southwest Minzu University, Chengdu, China; 2 School of Education, Sichuan Institute of Industrial Technology, Deyang, China; 3 School of Marxism, Shanghai University of International Business and Economics, Shanghai, China; 4 School of Marxism, Sichuan Institute of Industrial Technology, Deyang, China

**Keywords:** Zang-Qiang-Yi Corridor, ethnic minority residents, marriage intention, Andersen’s behavioral model, predisposing factors, enabling factors, demand factors

## Abstract

**Background:**

With the decline of the global fertility rate, the concepts of marriage and family formation have become important issues of societal concern.

**Methods:**

This paper employs Andersen’s behavioral model to examine the influence of predisposing factors (gender, age, residential place, religious belief), enabling factors (housing numbers, floating population), and demand factors (self-efficacy, family communication, intimate partner violence, anxiety, depression) on the marriage intention of ethnic minority residents in the Zang-Qiang-Yi Corridor.

**Results:**

The study finds that the marriage intention of ethnic minority residents in the Zang-Qiang-Yi Corridor significantly varies by gender, age, residential place, housing numbers, religious belief, and floating population status, but not by education level. Among these factors, demand factors (self-efficacy, family communication, intimate partner violence, anxiety, depression) have the most significant impact on marriage intention. Predisposing factors (gender, age, residential place, religious belief) and enabling factors (housing numbers, floating population) also have a certain degree of impact.

**Conclusion:**

These findings suggest that interventions targeting ethnic minority residents in the Zang-Qiang-Yi Corridor should consider a multifaceted approach, addressing both psychological and social determinants of marital intentions to improve marital outcomes.

## 1 Introduction

With the decline of the global fertility rate, the concepts of marriage and family formation have become important issues of societal concern. The level of marriage rate is closely related to marriage intention (Kuo and Raley, 2016; Raymo et al., 2021). Marriage intention refers to an individual’s expected state of mind regarding whether or not to enter into marriage. Traditionally, marriage has been regarded as a necessary milestone in one’s life, representing a new stage of personal development. However, in recent years, with societal development, gender equality, and the rise of values emphasizing freedom and openness, the trend of non-traditional and diverse marriage intentions has increased (Surkyn and Lesthaeghe, 2004). Some people argue that “Marriage is the tomb of love” a view often rooted in the belief that marriage restricts personal freedom and emotional expression. This perspective has led many to question the necessity of marriage, with some even arguing that the benefits of being single outweigh those of marriage. They see the commitment of marriage as a bondage and an invisible killer of freedom and happiness (Stanley et al., 2006). According to Life-Course Theory (Elder, 1998), entry into marriage is one of the most salient “life events” preceding fertility. Higher marriage intention increases the likelihood of entering marriage on time or even earlier, thereby raising the probability of childbearing within the reproductive span. Moreover, the Second Demographic Transition framework (Surkyn and Lesthaeghe, 2004) posits that, in low-fertility societies, marriage is no longer the sole prerequisite for childbearing; nevertheless, the sequence “marriage intention → actual marriage → fertility” still amplifies or buffers overall fertility levels. When marriage intention declines, even if non-marital births exist, they cannot fully compensate for fertility losses caused by delayed marriage. Using panel data from 37 countries, Myrskylä et al. (2013) found that, in nations with a total fertility rate (TFR) below 1.8, every one-per-mill decline in the marriage rate corresponded to an average TFR decrease of 0.02, demonstrating that the marital gateway remains a critical regulator of fertility in low-fertility contexts.

In the 1980s, Chinese scholar Fei introduced a new ethnological concept, the ethnic corridor, based on the research findings in the field of ethnology over many years (Li, 2005). The ethnic corridor is a summary of the historical spatial form that emerged under the special natural and historical conditions in China. It is an academic concept based on knowledge derived from local empirical data. In the early 1990s, Li (1995) first defined the ethnic corridor as the route along which a certain ethnic group migrates or flows outward over a long period, following a particular natural environment such as a river or mountain. In this corridor, there must be many historical and cultural deposits of the nation or ethnic group. Zhou and Zhou (2023) argue that the ethnic corridor is the boundary between the center of civilization and the contact zone. The essential feature of the ethnic corridor is its boundary nature. In the Zang-Qiang-Yi Corridor, where many ethnic groups live together, ethnic intermarriage has a long history. After the founding of New China, especially after the reform and opening up, ethnic intermarriage has become increasingly common (Ma, 2004). The wide range of ethnic intermarriage in the Zang-Qiang-Yi Corridor has effectively promoted exchanges between ethnic groups, improved ethnic relations, and fostered ethnic unity. It has also cultivated a strong sense of community among the Chinese nation (Wang, 2017; Zhang, 2022).

Research has indicated that marriage intention is influenced by a variety of factors, including gender, place of residence, education level, number of housing units, floating population status, social network size, self-efficacy, family communication, intimate partner violence, anxiety, and depression. According to Andersen’s behavioral model of health services use (Andersen, 1968), these factors can be categorized into predisposing factors, enabling factors, and demand factors.

### 1.1 Predisposing factors

Predisposing factors primarily refer to the characteristics that individuals possess, which may facilitate or hinder their willingness to enter into marriage. These factors can be divided into demographic factors and social structural factors. The former includes gender, place of residence, etc., while the latter mainly includes education level among others. Studies have shown that men generally have a higher marriage intention than women (Gove and Hughes, 1979; Oppenheimer, 1997; Blair and Madigan, 2020). Urban residents generally have lower marriage intention than their rural counterparts. Specifically, female urban residents have lower marriage intention than rural women with similar backgrounds. However, there is no significant difference in marital intention across different education levels (Kuo and Raley, 2016).

### 1.2 Enabling factors

Enabling factors include resources available to the family and community. Family resources refer to family income, deposits, property, floating population status, and other related conditions. Community resources include the accessibility of services in the community, social networks, and other community resources. Previous studies have shown that urban residents with low income levels have a higher marriage intention but are also more likely to divorce (Grinstein-Weiss et al., 2011). There is a positive correlation between real estate ownership and marriage intention. Assets such as self-owned housing have an important impact on the likelihood of marriage or divorce. Residents with real estate have a higher marriage intention, while couples without real estate are more likely to choose to delay marriage (Grinstein-Weiss et al., 2011). The instability associated with floating population status has slowed down people’s marriage intention to a certain extent. Additionally, residents with more harmonious family relationships and larger social networks tend to have a higher intention to marry (Lee et al., 2021).

### 1.3 Demand factors

Demand factors can be divided into two parts: one is subjective needs, such as self-efficacy and self-rated health status; the other is objective needs, such as family communication, anxiety, and depression. Studies have shown that individuals who have a stronger belief in their ability to become partners are more likely to be willing to establish long-term relationships, and thus have a higher marriage intention (Brooks-Gunn and Furstenberg, 1989). The more communication there is between family members, the more harmonious the family relationship, and the stronger their marriage intention. College students with harmonious family relationships tend to have a higher marriage intention, while those with discordant family relationships have a lower marriage intention (Wallerstein and Lewis, 2004). Excessive anxiety and depression are more likely to make individuals feel inferior and less willing to fall in love and get married (Loewenstein et al., 1981; Lynch, 2017).

Marriage is the union of two families, and it often requires individuals to sacrifice personal autonomy and take on family responsibilities (Yeung and Lu, 2023). Therefore, marrying late or not marrying at all has become another option for life planning (An et al., 2022). In the existing research, there is limited literature on the marriage intention and behavior of ethnic minority residents, and even less research focuses on the marriage intention of ethnic minority residents in the Zang-Qiang-Yi Corridor. The few studies that do exist are primarily limited to specific groups of people, such as college students and potential homebuyers (Xie and Hong, 2022), indicating a fragmented nature of these studies. Systematic research on the marriage intention of ethnic minority residents in the Zang-Qiang-Yi Corridor is still lacking.

### 1.4 Marriage intentions of Zang, Qiang, and Yi ethnic minority residents

In Zang culture, religion is not only a source of spiritual sustenance but also has a profound influence on marriage concepts (Childs, 2001). For example, Buddhism emphasizes cause and effect as well as fate, which makes Zang more inclined to follow traditions and religious doctrines in their marriage choices, thus influencing their marriage intentions. However, with the development of society, the marriage intentions of modern Zang youth are significantly influenced by education level, career development, and personal emotions. Increasingly, Zang youth are choosing their marriage partners independently (Chen et al., 2023). In urban and highly educated groups, marriage intentions are more focused on personal happiness and emotional satisfaction. Despite this increasing autonomy, family opinions still play an important role in marriage choices. The views of elders and family expectations continue to exert significant influence on their marriage decisions.

Historically, Qiang marriages were governed by the adage “in heaven, the Thunder God rules; on earth, the maternal uncle decides,” giving the uncle ultimate veto power and subordinating young people’s autonomy to clan interests (Yin, 2020). In recent decades, the spread of compulsory education and the rise of labor migration have increased youths’ economic contributions to the household, eroding the absolute authority of the maternal uncle and parents. When individuals perceive that education, skills, and earnings can shift their bargaining power in the “matching doors and households” calculus, their confidence in “choosing a spouse independently and sustaining the marriage” (i.e., self-efficacy) grows, thereby raising marriage intention. In short, the loosening of traditional authority has created institutional space for self-efficacy to be activated.

Before the founding of the People’s Republic, the Yi clan system imposed strict status boundaries on marriage; marrying within one’s rank was deemed essential for safeguarding family honor and preserving exclusive access to resources (Liu, 2025). The quantity of family housing not only signaled economic strength but also served as a visible marker of “matching doors” within the clan network. Families with more houses enjoyed higher prestige and their children held privileged access to the intra-clan marriage market, whereas those lacking property risked downward matches or delayed nuptials, perpetuating an intergenerational cycle of “poverty—housing shortage—marriage squeeze.” With the expansion of schooling and the ubiquity of labor migration, Yi youth now accumulate capital in cities like Chengdu and Kunming. Real estate acquired in urban areas can be mortgaged or rented out, offering migrants a feasible “buy first, marry later” trajectory: urban property acts as collateral for betrothal gifts when they return home, while a new rural house provides the post-wedding living space. Hence, greater housing assets enhance young people’s sense of control over future married life and, in turn, elevate their willingness to marry.

### 1.5 The present study

Since its introduction in 1968, Andersen’s behavioral model has been extended to a variety of non-medical behaviors, including mental-health service use (Fortin et al., 2018), participation in premarital education programs (Park et al., 2022), elder-care decisions (Ning et al., 2022), and training needs for grandparent caregivers (Song et al., 2024). Its three-dimensional structure—predisposing, enabling, and need factors—posits that any behavioral intention is jointly shaped by “ascribed characteristics, resource conditions, and subjective/objective needs.” Although marriage is not a medical act, the decision to marry can be framed as “a behavioral inclination formed under specific structural and resource conditions to satisfy intimacy and family needs.” Thus, the model is structurally isomorphic. The purpose of this study is to elucidate the level of marriage intention and the differences therein among ethnic minority residents in the Zang-Qiang-Yi Corridor through quantitative analysis. Based on the Andersen’s behavioral model (Andersen, 1968), the study systematically explores the key factors influencing the marriage intention of ethnic minority residents in the Zang-Qiang-Yi Corridor. A comprehensive model is established using multiple linear regression analyses to assess the relative contributions of different factors to marriage intention and to determine their predictive capacity. By conducting this research in ethnic minority areas, the study provides new perspectives and empirical data to support the understanding of the social and psychological mechanisms underlying marital behavior.

Based on the above, the following hypotheses are proposed:

*H1*: There are significant differences in marriage intentions among residents with different genders, floating population, religious belief, residential place, social-network sizes, and housing numbers.

*H2*: Gender, residential place, and religious belief are positively correlated with marriage intention.

*H3a*: Housing numbers are positively correlated with marriage intention.

*H3b*: Floating population status is negatively correlated with marriage intention.

*H4a*: Self-efficacy and family communication are positively correlated with marriage intention.

*H4b*: Intimate partner violence, anxiety, and depression are negatively correlated with marriage intention.

*H5*: Marriage intention differs significantly across ethnic groups.

## 2 Methods

### 2.1 Study population and design

The questionnaires for this study were collected between June and August 2024. A total of 630 participants aged 20 and above took part in this study. The research team used a simple random sampling technique to recruit participants from the Zang-Qiang-Yi Corridor. Participants were invited to take part in the survey through a web link. Participants did not receive payment for their participation in the survey. A pilot study was conducted with 39 participants before the main data collection.

### 2.2 Measures

#### 2.2.1 Sociodemographic characteristics

The demographic variables include seven items for gender, residential place (rural or urban), education level, floating population status, housing numbers, and social network size. The social network size question is as follows: On a typical day in the past month, how many non-relatives, other than family members and relatives, did you have contact with, regardless of the means of contact?

#### 2.2.2 Marriage intention

Marriage intention was measured using a self-reported question. The question was specifically, “Is your intention to get married?” Responses were measured on a scale ranging from 0 to 100, where a higher score indicates a higher intention to get married. Here, 0 indicates no intention to get married, while 100 indicates the highest possible intention.

#### 2.2.3 Family communication

Family communication was measured using the Family Communication Scale developed by Barnes and Olson (1985). The scale consists of 10 questions, and each question is scored on a 5-point Likert scale. Higher scores indicate better family communication. The Cronbach’s alpha coefficient for this scale in this study was 0.965, indicating excellent internal consistency.

#### 2.2.4 Self-efficacy

The short version of the General Self-Efficacy Scale developed by Chen et al. (2001) was used. The original scale consists of 10 items, while the short version used in this study contains 3 items. Specific items included: (1) When faced with difficult tasks, I am confident that I will be able to complete them; (2) I will be able to successfully overcome many challenges; (3) I am confident that I will be able to perform many different tasks effectively. Each item was scored on a 5-point Likert scale, where higher scores indicate higher self-efficacy. The scale’s validity indicators were well-fitted, with *χ*^2^/df = 2.29, RMSEA = 0.027, CFI = 0.992, TLI = 0.989, and SRMR = 0.031. These indicators suggest that the scale has good construct validity. The Cronbach’s alpha coefficient for the scale in the current study was 0.914, indicating high internal consistency.

#### 2.2.5 Depression

The Patient Health Questionnaire developed by Kroenke and Spitzer (2002) and revised by Bian et al. (2009) was used to measure the level of depression in individuals. The scale consisted of nine items, each of which was rated on a 4-point Likert scale, where higher scores indicate higher levels of depression. The Cronbach’s alpha coefficient for the scale in this study was 0.933, indicating high internal consistency.

#### 2.2.6 Intimate partner violence

Based on the literature related to domestic violence attitudes, intimate partner violence attitudes, and the Partner Violence Attitude Scale (Huang et al., 2007; Li et al., 2020; Xie et al., 2022), the self-administered Intimate Partner Violence Scale measures intimate partner violence. The scale consists of two dimensions: physical violence and mental violence, with five items. Each item was scored on a 5-point Likert scale, where higher scores indicate higher levels of intimate partner violence. The scale’s validity indicators were well-fitted, with χ^2^/df = 2.78, RMSEA = 0.035, CFI = 0.990, TLI = 0.982, and SRMR = 0.037. These indicators suggest that the scale has good construct validity. The Cronbach’s alpha coefficient for the scale in the present study was 0.946, indicating high internal consistency.

#### 2.2.7 Anxiety

The Generalized Anxiety Rating Scale (GARS) developed by Spitzer et al. (2006) and translated and revised by He et al. (2010) was used to measure the anxiety level of individuals. The scale consists of seven items, each of which is rated on a 4-point Likert scale. Higher scores indicate higher levels of anxiety. The Cronbach’s alpha coefficient for this scale in the current study was 0.958, indicating high internal consistency.

### 2.3 Statistical analysis

SPSS 24.0 was used for statistical description and inference of the data. Harman’s single-factor test was used to assess common method bias using *χ*^2^ and *t*-tests. The measurement items of five variables—self-efficacy, family communication, intimate partner violence, anxiety, and depression—were subjected to factor analysis, and five factors with eigenvalues greater than 1 were extracted. The results showed that the largest factor variance contribution was 37.77%, which was lower than the critical value of 40%, ruling out the problem of common method bias. Based on Andersen’s behavioral model as the theoretical framework, a stepwise multiple linear regression model was used for multifactor analysis, with predisposing factors as control variables, to construct the baseline Model I. On the basis of Model I, enabling factors were included to construct Model II, and on the basis of Model II, demand factors were included to construct Model III, with the significance level *α* = 0.05. The models are as follows:


$$\text{Model}\;I:\mathrm{Y1}=\beta_{0}+\beta_{1}X_{\text{predisposing factors}}$$

$$\text{Model}\;\mathrm{II}:\mathrm{Y2}=\beta_{0}+\beta_{1}X_{\text{predisposing factors}}+\beta_{2}X_{\text{enabling factors}}$$

$$\begin{array}{l} \text{Model}\;\mathrm{III}:\mathrm{Y3}=\beta_{0}+\beta_{1}X_{\text{predisposing factors}}+\beta_{2}X_{\text{enabling factors}}+\\ \beta_{3}X_{\text{demand factors}} \end{array}$$

## 3 Results

### 3.1 Sample sociodemographic characteristics

The sociodemographic characteristics of the sample are presented in Table 1. Of the 630 participants, 239 (37.94%) were Zang, 87 (13.81%) Qiang, 181 (28.73%) Yi, and 123 (19.52%) belonged to other ethnic minorities. Participants aged 20–25 accounted for 195 (30.95%), those aged 26–30 for 172 (27.30%), aged 31–35 for 146 (23.17%), and 117 (18.58%) were 36 or older. There were 238 males (37.78%) and 392 females (62.22%). Educational attainment was distributed as follows: 37 participants (5.87%) had completed junior high school or below, 120 (19.05%) high school or secondary school, 171 (27.14%) junior college, 225 (35.72%) a regular college (bachelor’s) course, and 77 (12.22%) postgraduate studies. With respect to migration status, 271 individuals (40.02%) were non-floating residents, whereas 359 (57.98%) were classified as floating population. Residential location showed that 433 participants (68.73%) lived in cities or towns, and 197 (31.27%) in rural districts. The required sample size was determined *a priori* using G*Power 3.1.9.7. Assuming a medium effect size *f^2^* = 0.10, significance level *α* = 0.05, statistical power 1 – *β* = 0.90, and nine predictors, the software indicated a minimum sample of 149 cases. The actual sample of 630 participants therefore exceeds this requirement.

**Table 1:** Sociodemographic characteristics of the sample (*n* = 630).

Characteristics of participants	Variables	Number (%)
Ethnic	Zang	239 (37.94%)
	Qiang	87 (13.81%)
	Yi	181 (28.73%)
	Other ethnic minority	123 (19.52%)
Age	20–25	195 (30.95%)
	26–30	172 (27.30%)
	31–35	146 (23.17%)
	≥36	117 (18.58%)
Educational level	Junior high school and below	37 (5.87%)
	High school or secondary school	120 (19.05%)
	Junior college	171 (27.14%)
	Regular college course	225 (35.72%)
	Postgraduate	77 (12.22%)
Floating population	No	271 (40.02%)
	Yes	359 (57.98%)
Genders	Male	238 (37.78%)
	Female	392 (62.22%)
Residential place	Cities and Towns	433 (68.73%)
	Rural district	197 (31.27%)
Social network size	0	118 (18.73%)
	1–4	218 (34.60%)
	5–9	126 (20.00%)
	10–19	86 (13.65%)
	20–49	35 (5.56%)
	50–99	27 (4.29%)
	≥100	20 (3.17%)
Housing numbers	No	101 (16.03%)
	1 set	330 (52.38%)
	2 sets	127 (20.16%)
	≥ 3 sets	
Religious belief	Yes	167 (26.51%)
	No	463 (73.49%)

### 3.2 Analysis of differences in marriage intention among ethnic minority residents in the Zang-Qiang-Yi Corridor

There are statistically significant differences in the marriage intention among ethnic minority residents in the Zang-Qiang-Yi Corridor in terms of gender (*p* < 0.001), age (*p* < 0.05), floating population (*p* < 0.05), residential place (*p* < 0.01), social network size (*p* < 0.05), housing numbers (*p* < 0.05), and religious belief (*p* < 0.001), and there is no significant difference in terms of education level (*p* > 0.05). Female, cities and towns, those with worse social network relationships, smaller social networks, fewer housing numbers, no religious belief, and floating population status have a relatively lower intention to get married, as shown in Table 2.

**Table 2:** One-way analysis of the marriage intention score of ethnic minority residents in the Zang-Qiang-Yi Corridor (*n* = 630).

Characterization		Score	*t/F*	*p*-value
Genders	Male	53.16 ± 28.35	26.05	<0.001
	Female	41.86 ± 26.05		
Educational level	Junior high school and below	48.00 ± 25.78	0.12	0.974
	High school or secondary school	46.58 ± 27.90		
	Junior college	44.41 ± 28.22		
	Regular college course	46.15 ± 27.51		
	Postgraduate	45.14 ± 26.00		
Floating population	No	49.02 ± 26.72	5.47	<0.05
	Yes	43.88 ± 27.88		
Age	20–25	47.12 ± 28.04	3.48	<0.05
	26–30	48.19 ± 28.65		
	31–35	42.42 ± 23.05		
	≥36	33.28 ± 21.43		
Residential place	Cities and Towns	44.19 ± 28.94	6.99	<0.01
	Rural district	50.40 ± 23.46		
Social network size	0 people	38.62 ± 25.75	2.29	<0.05
	1–4 people	46.51 ± 26.32		
	5–9 people	46.87 ± 28.83		
	10–19	49.33 ± 27.62		
	20–49	49.46 ± 28.27		
	50–99	52.55 ± 25.91		
	≥100	53.38 ± 33.30		
Housing numbers	No	43.71 ± 22.69	3.18	<0.05
	1 set	44.34 ± 23.85		
	2 sets	47.97 ± 33.29		
	≥ 3 sets	54.50 ± 35.49		
Religious belief	Yes	57.40 ± 30.48	3.59	<0.001
	No	44.79 ± 26.81		

### 3.3 Multifactorial analysis of differences in marriage intention among ethnic minority residents in the Zang-Qiang-Yi Corridor

A linear regression model was constructed with marriage intention as the dependent variable, gender and residential place as the predisposing factors screened after univariate analysis, housing numbers and floating population as the enabling factors, and self-efficacy, family communication, intimate partner violence, anxiety, and depression as the demand factors, with predisposing factors, enabling factors, and demand factors as the independent variables. Before inclusion in the model, a collinearity test was performed on the influencing factors, and the Variance Inflation Factor (VIF) was less than 5, indicating no multicollinearity problem. The results of Model I show that gender (*β* = −0.206, *p* < 0.001), age (*β* = −0.129, *p* < 0.001), residential place (*β* = 0.084, *p* < 0.05) and religious belief (*β* = 0.161, *p* < 0.001) were influencing factors of ethnic minorities’ marriage intention in the Zang-Qiang-Yi Corridor. Model II added the enabling factors, and housing numbers (*β* = 0.132, *p* = 0.001) and floating population (*β* = −0.158, *p* < 0.001) were identified as influencing factors of ethnic minorities’ marriage intention in the Zang-Qiang-Yi Corridor. Model III added the demand factors, the results showed that self-efficacy (*β* = 0.129, *p* < 0.001) and family communication (*β* = 0.101, *p* = 0.009) positively predicted ethnic minorities’ marriage intention in the Zang-Qiang-Yi Corridor, while intimate partner violence (*β* = −0.094, *p* = 0.020), anxiety (*β* = −0.130, *p* = 0.007), and depression (*β* = −0.105, *p* = 0.029) negatively predicted ethnic minorities’ marriage intention in the Zang-Qiang-Yi Corridor. Model III had the strongest explanatory power overall, at 28.885%. The effects of the factors influencing ethnic minorities’ marriage intention in the Zang-Qiang-Yi Corridor, in descending order, were: Gender (*β* = −0.157) > Residential place (*β* = 0.155) > Housing numbers (*β* = 0.148) > Religious belief (*β* = 0.135) > Anxiety (*β* = −0.130) > Self-efficacy (*β* = 0.129) > Floating population (*β* = −0.112) > Depression (*β* = −0.105) > Family Communication (*β* = 0.101) > Intimate partner violence (*β* = −0.094) > Age (*β* = −0.080). The details are shown in Table 3.

**Table 3:** Multi-factor linear regression analyses of the factors influencing ethnic minorities’ marriage intention in the Zang-Qiang-Yi Corridor.

Factor	Regression coefficient	Standard error	Standardized regression coefficient	*t*	*p*	*R* ^2^	Δ*R*^2^	*F*
Predisposing factors						0.083	0.077	14.178
Gender	−11.649	2.173	−0.206	−5.360	<0.001			
Age	−4.106	1.240	−0.129	−3.311	<0.001			
Residential place	5.062	2.302	0.084	2.199	0.028			
Religious belief	14.379	3.482	0.161	4.129	<0.001			
Enabling factors						0.121	0.112	17.267
Gender	−11.107	2.134	−0.196	−5.204	<0.001			
Age	−3.384	1.225	−0.107	−2.761	0.006			
Residential place	7.891	2.380	0.132	3.314	<0.001			
Religious belief	12.427	3.442	0.140	3.610	<0.001			
Housing numbers	4.184	1.269	0.132	3.297	0.001			
Floating population	−8.776	2.096	−0.158	−4.187	<0.001			
Demand factors						0.252	0.238	28.885
Gender	−8.863	1.999	−0.157	−4.433	<0.001			
Age	−2.549	1.138	−0.080	−2.239	0.026			
Residential place	9.283	2.220	0.155	4.181	<0.001			
Religious belief	12.026	3.191	0.135	3.769	<0.001			
Housing numbers	4.719	1.178	0.148	4.006	<0.001			
Floating population	−6.225	1.961	−0.112	−3.517	0.002			
Self-efficacy	4.319	1.228	0.129	−3.517	<0.001			
Family communication	3.291	1.259	0.101	2.614	0.009			
Intimate partner violence	−2.404	1.032	−0.094	−2.330	0.020			
Anxiety	−4.591	1.689	−0.130	−2.178	0.007			
Depression	−4.025	1.844	−0.105	−2.183	0.029			

### 3.4 Heterogeneity of marriage intention across ethnic groups

We conducted separate regression analyses for Zang, Qiang, Yi, and other ethnic minorities; the results are reported in Table 4. Zang: residential place (*B* = 9.679, *t* = 2.180, *p* < 0.05), religious belief (*B* = 18.926, *t* = 4.538, *p* < 0.001), family communication (*B* = 6.562 = 3.134, *p* < 0.001), and depression (*B* = −5.517, *t* = −1.979, *p* < 0.05) significantly predicted marriage intention. Qiang: gender (*B* = 11.131, *t* = −1.996, *p* < 0.05) and self-efficacy (*B* = 10.228, *t* = 2.708, *p* < 0.01) were significant predictors. Yi: gender (*B* = −11.572, *t* = −2.990, *p* < 0.01), housing numbers (*B* = 7.924, *t* = 2.990, *p* < 0.01), intimate partner violence (*B* = −6.109, *t* = −2.815, *p* < 0.001), and depression (*B* = −9.512, *t* = −2.349, *p* < 0.05) significantly influenced marriage intention. Other ethnic minorities: gender (*B* = −12.223, *t* = −2.866, *p* < 0.01), residential place (*B* = 13.445, *t* = 3.051, *p* < 0.01), Housing numbers (*B* = 6.353, *t* = 2.706, *p* < 0.01) and anxiety (*B* = −13.971, *t* = −4.534, *p* < 0.001) were the significant predictors. These results demonstrate significant heterogeneity in marriage intention across the four ethnic groups.

**Table 4:** Heterogeneity analysis of marriage intention among ethnic minority residents in the Zang-Qiang-Yi Corridor.

Factor	Zang	Qiang	Yi	Other ethnic
Constant	2.90 (0.168)	−8.780 (−0.297)	77.933** (2.906)	20.079 (0.907)
Gender	−2.489 (−0.699)	−11.131* (−1.996)	−11.572** (−2.990)	−12.223** (−2.866)
Age	−1.898 (−1.070)	−5.973 (−1.766)	−1.595 (−0.632)	−0.397 (−0.182)
Residential place	9.679* (2.180)	6.524 (1.123)	7.630 (1.556)	13.445** (3.051)
Religious belief	18.926** (4.538)	9.905 (1.362)	−21.219 (−1.392)	5.708 (0.489)
Housing numbers	2.114 (1.197)	3.521 (0.970)	7.924** (2.990)	6.353** (2.706)
Floating population	−5.267 (−1.455)	−11.489 (−1.880)	−4.681 (−1.218)	−5.165 (−1.254)
Self-efficacy	1.301 (0.640)	10.228** (2.708)	3.834 (1.568)	4.092 (1.671)
Family communication	6.562** (3.134)	6.820 (1.701)	−2.936 (−1.138)	3.794 (1.574)
Intimate partner violence	0.492 (0.322)	0.518 (0.127)	−6.109** (−2.815)	−3.318 (−1.501)
Anxiety	−3.368 (−1.225)	5.026 (0.926)	2.750 (0.732)	−13.971** (−4.534)
Depression	−5.517* (−1.979)	−5.823 (−0.976)	−9.512* (−2.349)	2.983 (0.767)
Sample size	239	87	181	123
*R* ^2^	0.243	0.364	0.244	0.492
Adjusted *R* ^2^	0.207	0.271	0.195	0.442
*F*	*F* (11,227) = 6.640, *p* = 0.000	*F* (11,75) = 3.902, *p* = 0.000	*F* (11,169) = 4.972, *p* = 0.000	*F* (11,111) = 9.779, *p* = 0.000

* *p* < 0.05 ** *p* < 0.01 *** *p* < 0.001.

## 4 Discussion

### 4.1 Implications of the differences in marriage intention among ethnic minority residents

The findings of this study reveal significant variations in marriage intention among ethnic minority residents in the Zang-Qiang-Yi Corridor, H1 was verified. The lower marriage intention observed among females, urban residents, floating population groups, those with fewer housing resources, and those with smaller social network sizes highlights the complex interplay of socio-economic and cultural factors in shaping marital behavior.

The lower marriage intention among females compared with males may be attributed to the changing social roles and increasing economic independence of females in ethnic minority areas (Cislaghi and Heise, 2020). This trend aligns with broader global patterns where women’s empowerment and education often lead to delayed and reduced marriage intentions. Additionally, cultural expectations and gender norms in these communities might also play a role, which warrants further qualitative exploration. The higher marriage intention among rural residents could be due to stronger traditional values and social pressures to get married within rural communities (Qing, 2023). In contrast, urban residents may face more diverse lifestyle choices, higher living costs, and greater exposure to modern values that diminish the urgency and desire to marry. Floating populations, often migrating for work or education, face unique challenges such as instability, social isolation, and cultural disconnection (Shao et al., 2023). These factors likely contribute to a lower marriage intention among them, as they may prioritize economic stability and personal development over immediate family formation. The influence of social network size and housing numbers suggests that social support and material conditions are crucial determinants of marriage intention (Özşahin, 2024). Smaller social networks may limit access to potential partners and social approval, while limited housing resources could indicate economic constraints that deter marriage.

### 4.2 Multifactorial influences on marriage intention

The multifactorial analysis using the Andersen’s behavioral model provides a comprehensive framework for understanding the key determinants of marriage intention among ethnic minority residents. The model integrates predisposing factors (gender, residential status), enabling factors (housing numbers, floating population status), and demand factors, including self-efficacy, family communication, intimate partner violence, anxiety, and depression, H2a, H2b, H3a, and H3b were verified.

Gender and residential place emerged as significant predisposing factors, indicating that inherent demographic characteristics and cultural contexts have a strong influence on marriage intention (Breger and Hill, 2021). The gender disparity underscores the need for targeted interventions to address the unique challenges faced by women in ethnic minority areas, such as enhancing their socio-economic status and overcoming cultural barriers. The significant negative effect of age is consistent with the expectations of life course theory (Elder, 1998): as individuals grow older, they gradually deviate from the societal “marriage-on-time” clock, the structure of marriage opportunities contracts, and marriage intention declines accordingly. Housing numbers and floating population status were identified as key enabling factors. These findings highlight the importance of material resources and stability in shaping marital decisions (Kravdal et al., 2023). Policies aimed at improving housing conditions and providing support for floating population groups could enhance marriage intention and overall well-being in these communities. The significant role of self-efficacy and family communication in positively correlated marriage intention suggests psychological empowerment and supportive family environments are crucial (Çoban, 2022). Conversely, intimate partner violence, anxiety, and depression negatively correlated marriage intention, indicative of the critical concerns of mental health and relationship quality.

After controlling for predisposing and enabling factors, the inclusion of need factors increased the explained variance from 10.1 to 22.9%, confirming their independent contribution in the context of marriage intention. Although constructs such as self-efficacy and anxiety differ semantically from the original “health-care need” domain, they serve the same functional role within the Andersen framework—namely, as significant predictors of behavioral intention.

### 4.3 Discussion of ethnic heterogeneity in marriage intention

As shown in the ethnicity-specific regressions in Table 4, Zang, Qiang, Yi and other minority groups display marked heterogeneity in the mechanisms shaping marriage intention, corroborating H5. These differences reflect both inter-ethnic cultural variation and the distinct roles played by socio-psychological resources across groups.

Marriage intention among Zang is jointly shaped by four factors—residential place → religious belief → family communication → depression. Among them, religious belief exerts the largest effect size, confirming the centrality of religion in Zang society: devout adherence is usually accompanied by more traditional marital norms and stronger familistic values, which significantly elevate the propensity to marry (Childs, 2001). The significant positive effect of residential place indicates that individuals living in traditional pastoral areas or in close proximity to monasteries, where religion and community exert a dual influence, exhibit higher marriage intention. High-quality family communication also significantly enhances the willingness to marry, reflecting the emphasis on emotional connectedness within the Zang “extended family” culture. Conversely, the negative effect of depression is consistent with prior research, demonstrating that mental-health risks can directly erode confidence in future marital life.

Qiang sample. Only gender and self-efficacy reach significance, yielding a comparatively parsimonious structure. The negative gender coefficient indicates that Qiang women display significantly lower marriage intention than men, a pattern likely linked to rising female educational attainment and heightened economic independence in Qiang areas (Li and Geng, 2023). The positive effect of self-efficacy shows that the stronger one’s perceived capability, the greater the inclination to enter marriage and assume family responsibilities. Notably, neither religious nor family factors emerge as significant for the Qiang, suggesting that the regulatory force of religion and kin networks may have weakened during modernization, while individual-level psychological resources have gained relative salience.

The Yi regression yields the most complex configuration, encompassing four key predictors—gender → housing assets → intimate partner violence → depression. The gender effect parallels that of the Qiang, with Yi women manifesting significantly lower marriage intention. The positive effect of housing stock underscores the importance of economic capital in the Yi marriage market: in Yi communities, housing is not only a prerequisite for marriage but also a visible marker of the groom’s family socioeconomic standing (Liu, 2025). The negative effect of intimate partner violence indicates that anticipated or experienced violence markedly reduces women’s willingness to marry, aligning with the recent rise in female rights awareness and heightened expectations for marital quality among the Yi. The negative effect of depression reconfirms the pervasive influence of mental health on marital decision-making.

### 4.4 Strengths and limitations of the study

The study employed a robust quantitative approach, utilizing multiple linear regression analyses to identify and quantify the relative contributions of various factors influencing marriage intention. The application of the Andersen’s behavioral model provides a structured framework for understanding the complex interplay of predisposing, enabling, and demand factors. The findings offer valuable insights into the socio-economic and psychological mechanisms underlying marital behavior in ethnic minority areas, contributing to the existing literature.

The study sample comprises ethnic-minority residents aged 20 and above in the four prefectures of the Zang–Qiang–Yi Corridor, where the economy is dominated by farming-pastoralism, tourism, and migrant labor. Consequently, the findings should not be directly generalized to ethnic minorities in eastern coastal cities, cross-border communities, or groups with a higher degree of nomadism. Web-based convenience sampling was used, likely excluding individuals without smartphones or internet access (e.g., residents in remote pastoral areas and older adults), resulting in an over-representation of younger, urban, and more-educated participants. Additionally, voluntary participation may introduce self-selection bias toward individuals with a heightened interest in marriage-related issues. The study relied exclusively on quantitative data, which may limit the depth of understanding of the underlying cultural and contextual influences on marriage intention. Qualitative research could provide richer insights into the lived experiences and personal perceptions of ethnic minority residents. The sample was restricted to the Zang-Qiang-Yi Corridor, which may restrict the generalizability of the findings to other ethnic minority regions. Future research could explore broader geographic regions to enhance the applicability of the results. The study did not consider potential confounding variables such as religious beliefs, cultural practices, or historical contexts that could influence marriage intention. Further research incorporating these factors could provide a more nuanced understanding of marriage intention among ethnic minorities. Future research could also adopt a mixed-methods design, conducting in-depth interviews with Zang (Buddhist), Qiang (indigenous beliefs/Zang Buddhism), and Yi (Bimo-culture) communities to explore marriage rituals, dowry/bride-price norms, and kin-network expectations within their distinct religious–cultural contexts, thereby supplementing the cultural mechanisms that quantitative models cannot capture. This cross-sectional study design precludes establishing causal relationships between demand factors and marriage intentions. For instance, depression may suppress marriage intentions, while prolonged singlehood may exacerbate depressive levels. Future research employing longitudinal tracking or quasi-experimental designs (e.g., pre- and post-policy intervention comparisons) is recommended to clarify temporal relationships and causal pathways among variables.

### 4.5 Future research and practical implications

This study provides a foundation for further research into marriage intention in ethnic minority areas. Future studies could consider the following approaches: Conduct mixed-methods research to integrate quantitative findings with qualitative insights, thereby offering a more comprehensive understanding of the factors influencing marriage intention. Explore the role of cultural and religious factors in shaping marital behaviors, as these elements can significantly influence the attitudes and decisions of ethnic minority residents. Investigate the long-term impacts of marriage intention on social stability, economic development, and family structures in ethnic minority communities. Future studies should incorporate more refined indicators—such as “level of religious participation” “frequency of traditional rituals” and “preference for intra-ethnic marriage”—to examine the mediating or moderating roles of religious and cultural variables in marital decision-making.

From a practical standpoint, the findings suggest several policy and intervention recommendations:

*Gender Empowerment Programs*: Initiatives aimed at enhancing women’s economic independence and social status could help narrow the gender gap in marriage intention.

*Mental Health Support Programs*: Initiatives targeting mental health, particularly anxiety and depression, could improve overall well-being and positively impact marital decisions.

*Social and Economic Support Policies*: Policies addressing housing shortages and providing support for floating populations could create more conducive environments for marriage and family formation.

*Family and Relationship Education Programs*: Initiatives promoting healthy family communication and addressing intimate partner violence could foster more stable and supportive relationships, thereby promoting marriage intention.

## 5 Conclusion

The study concludes that the marriage intention of ethnic minority residents in the Zang-Qiang-Yi Corridor is influenced by the complex interplay of multiple factors. Among these, demand factors such as self-efficacy, family communication, intimate partner violence, anxiety, and depression have the most significant impact on individuals’ marriage intention. These factors likely influence individuals’ perceptions, attitudes, and decisions regarding marriage. Additionally, predisposing factors like gender and residential place, as well as enabling factors such as housing numbers and their status as part of the floating population, also play a role in influencing marriage intention. This suggests that a comprehensive approach should be considered in interventions to improve marital outcomes for ethnic minority residents in the Zang-Qiang-Yi Corridor, addressing both the psychological and social determinants of marital intention.

## Data Availability

The raw data supporting the conclusions of this article will be made available by the authors, without undue reservation.
